# Automating the extraction of information from a historical text and building a linked data model for the domain of ecology and conservation science

**DOI:** 10.1016/j.heliyon.2022.e10710

**Published:** 2022-10-04

**Authors:** Vatsala Nundloll, Robert Smail, Carly Stevens, Gordon Blair

**Affiliations:** a School of Computing and Communications, Lancaster University, Lancaster, UK; b Lancaster Environment Centre, Lancaster University, UK^1^1 Robert Smail worked at this organisation.

**Keywords:** Data extraction, Unstructured data, Semantic integration, Natural language processing, Machine learning, Ontologies

## Abstract

Data heterogeneity is a pressing issue and is further compounded if we have to deal with data from textual documents. The unstructured nature of such documents implies that collating, comparing and analysing the information contained therein can be a challenging task. Automating these processes can help to unleash insightful knowledge that otherwise remains buried in them. Moreover, integrating the extracted information from the documents with other related information can help to make more information-rich queries. In this context, the paper presents a comprehensive review of text extraction and data integration techniques to enable this automation process in an ecological context. The paper investigates into extracting valuable floristic information from a historical Botany journal. The purpose behind this extraction is to bring to light relevant pieces of information contained within the document. In addition, the paper also explores the need to integrate the extracted information together with other related information from disparate sources. All the information is then rendered into a query-able form in order to make unified queries. Hence, the paper makes use of a combination of Machine Learning, Natural Language Processing and Semantic Web techniques to achieve this. The proposed approach is demonstrated through the information extracted from the journal and the information-rich queries made through the integration process. The paper shows that the approach has a merit in extracting relevant information from the journal, discusses how the machine learning models have been designed to classify complex information and also gives a measure of their performance. The paper also shows that the approach has a merit in query time in regard to querying floristic information from a multi-source linked data model.

## 1 Introduction

Recent advances in technology offer scientists opportunities to procure and analyse increasing amounts of data. These data are largely in the form of structured datasets, that is, mainly in the form of field label/value pair formats. Nonetheless, textual documents remain the primary mode through which scientific findings are presented and communicated to the broader scientific community and the public more generally. Given the largely unstructured nature of these documents, it can be quite challenging when it comes to correlate, compare and analyse the findings from such textual sources. Moreover, a lot of the required information can also be frequently interspersed with information on other subjects, hence posing a problem to adequately extract meaningful information. This problem is further compounded with texts showing linguistic variations, which can be particularly seen in historical documents written in older versions of a particular language. Whilst the digital nature of these scientific texts means that they are easier to access and share, there are paper-based archival records that are also of interest to the scientific community and to historians especially. However, the valuable information sitting in these documents is more difficult to access and share. Moreover, manually reading a document and extracting any required information may be an easier route but only if the document is small-sized. This method quickly becomes tedious and time consuming with more importantly a large corpus of documents. These challenges have meant that, until relatively recently, scientists have accessed textual documents manually, and this has considerably limited the volume of texts that can be drawn upon and analysed by them.

Moreover, data stored in different formats or in disparate data sets contribute to the data heterogeneity problem (which is also one of the 4 V's of ‘big data’ namely ‘variety’ along with ‘volume’, ‘velocity’ and ‘veracity’). This data heterogeneity problem causes data to be silo-ed which poses a serious problem to domain specialists to move towards an integrated and more collaborative model of working. This siloing problem can be overcome by bringing data together in one place. However, this problem is further compounded with unstructured forms of data. With an increasing volume of textual data, either from documents or from social media websites, there is also a need to bring in data both from structured as well as unstructured sources.

Bringing these threads together, this paper investigates possible technological solutions to achieve data extraction and data integration in the scientific domain, with a particular focus on a case study in the field of ecology and conservation science. This breaks down into the following research questions:

1. To what extent can we extract information from unstructured historic texts?

2. To what extent can we integrate the extracted information with other information from a range of disparate sources?

3. To what extent can we then gain scientific insight from querying across the resultant integrated information model?

In this respect, there is an urgent need to seek automated methods drawing on a combination of Machine Learning and Natural Language Processing techniques to extract data and information from unstructured sources. In addition, Semantic Web techniques can be applied to bring together information from disparate sources and enable richer queries from multiple sources. In more detail, the paper looks at emerging computational technologies through a combination of Natural Language Processing, Machine Learning and the Semantic Web to devise an intelligent method to automate the extraction of empirical data from a historical text and to query this data from a unified model bringing together other related data sets. The aim is to make richer queries from this unified model that draws data from heterogeneous data sets. Natural Language Processing (NLP) is a field of study combining computer science and linguistics to study the interactions between machine and the human language. NLP helps the machine to understand the syntax and meaning of the human language. Machine Learning is a subset of Artificial Intelligence and is a study of computer algorithms to help train a machine to automatically learn through experience without being explicitly programmed. Semantic Web technologies enable to build data dictionaries and combine heterogeneous data sets into a linked data model.

We investigate the potential of such techniques through a case study, from the field of ecology and conservation science, on exploring the evolution of plant species over time in a region of the UK. The United Kingdom has a very strong tradition in the accurate observation of plants extending back to at least the seventeenth century. Consequently, there are extensive records which we can draw upon, including scientific Society Transactions and Proceedings, regional and national floras, scientific journals, letters, field notebooks and travel accounts. By enabling access to the empirical data contained within these sources, it is expected that a better understanding of the region past biodiversity can be revealed, thus allowing for more effective conservation strategies to be developed. Published over past three centuries ago, the Journal of Botany contains rich floristic information considered of valuable importance by conservation scientists and historians in the UK. However, it was only recently available in paper format, making the search for information a very tedious and time consuming process. This problem is further compounded by its dense nature, making this document very difficult to use. In our case study, we focus on the automated extraction of floristic information from the Journal of Botany. We believe that the application of the computational techniques mentioned above can automate the handling of complex data, bring together information gathered from various sources and enable easy access to ‘isolated’ information.

The paper is structured as follows: section 2 talks about some existing literature in this area; section 3 presents a case study on the extraction of floristic information; section 4 shows the methodology used; section 5 presents the results obtained; section 6 provides a discussion of the approach; and finally section 7 concludes the paper.

## 2 Related work

In the digital humanities, a variety of textual sources including travel writings (Donaldson et al., 2017), census records (Atkinson et al., 2017), newspapers and periodicals (Porter et al., 2018) have been interrogated using different techniques. Geographical Text Analysis (GTA) (Donaldson et al., 2017, Gregory and Donaldson, 2016) is one such research area having particular relevance in the extraction of historical flora from texts. This technique is used to uncover the geography of a term/set of terms from a textual source, enabling researchers to identify any empirical information and geospatial attributes recorded alongside the term. This is of particular relevance in textual sources where geographical context is important. To date, the methods used for examining historical texts have largely been rule-based. One such example would be as follows: find all locations first, and then find all mentions of plant names from the text surrounding a given location name. Such rule-based approaches have sometimes been described as a ‘top down’ approach to reveal patterns or relationships in the subject text (Jackson and Moulinier, 2007), frequently represented in the form of search lists, gazetteers, dictionaries and grammatical patterns. Although rule-based approaches have proven to be effective, they display a number of potential limitations, particularly when the text contains spelling variations. These variations impede the efficiency of the rule-based approaches which rely heavily on the integrity of the words. For example, some consulting texts covering a wide time span will likely result in spelling variations of location names that can have a detrimental effect on the accuracy of such rule-based techniques. Moreover, the language used in texts, especially in historical texts, can remain fluid thus giving rise to variations in the words. To identify place-names across a large volume of such texts will likely involve identifying multiple spellings variations while the tracing of plant species may require looking for multiple synonym names and then ‘mapping’ each synonym back to its current accepted name (Donaldson et al., 2017). These challenges trigger the need for more robust techniques such as word analysis, part-of-speech (POS) tagging and semantic analysis to handle the inconsistencies occurring as a result of spelling variations (Baron and Rayson, 2009, Lopresti, 2009, Butler et al., 2017).

Another pressing problem emerges during the digitisation of paper documents. There are many documents such as historical archives which are only in paper form. These documents need to be converted into a digital form before we can automatically extract any information from them. This digitization process is possible through software such as Optical Character Recognition (OCR) (Bennamoun and Mamic, 2012, pp. 199–220). Automated OCR is used extensively in digitising historical texts (Hitchcock, 2013) because it is less expensive and less time-consuming than manual digitisation. However, the accuracy of automated OCR software is variable with a percentage of characters and words being misidentified (Hitchcock, 2013, Tanner et al., 2009, Amelia, 2017). This is particularly relevant in historical texts where the print is often of poor quality or the paper has aged. Authors in (Lopresti, 2009) have shown that the false identification of characters using OCR software has the potential to lead to a ‘cascading effect’, where misidentified characters effect all subsequent analysis. This has a direct consequence on a proper rendering of the paper in digital form. Consequently, this impacts on the adequate interpretation of the language in the text using Natural Language Processing techniques such as word and sentence tokenisation, part-of-speech tagging (Lopresti, 2009, Tanner et al., 2009, Kettunen and Pääkkönen, 2016) in correctly interpreting text. Lately, the use of Machine Learning techniques has been gaining traction in building models that can be trained to recognise information entities from textual sources. The accuracy of the Machine Learning models determines whether they can also recognise spelling variations occurring in the entities.

The combination of Machine Learning and Natural Language Processing techniques to build models to interpret the human language, and use the models to extract relevant information from text is very compelling. A lot of research is currently being carried out using this combination of techniques in the field of data mining and information extraction. For instance, (Romanov et al., 2019) looks at automatically classifying text messages in Russian language using Machine Learning and Natural Language Processing methods (in particular sentiment classification - a field in Natural Language Processing). They tested different classifier algorithms, and found that classifiers based on Support Vector Machine could efficiently solve the problem of classifying short scientific texts. (Spasić et al., 2014) reviews the research literature on text mining, aiming to understand where text mining is being applied in cancer domains, which resources can enable to apply text mining of cancer-related information and to what extent Natural Language Processing methods can be used to structure information from free text reports. (Van Houtan et al., 2020) applies Machine Learning and Natural Language Processing techniques to extract and analyse explicit information from a large corpus of scientific abstracts in reintroduction biology. The authors have applied sentiment analysis to understand the positive or negative sentiments of the text. The paper shows that sentiment trends in these studies seem to show the successes and challenges for conservation biology. However, very few papers discuss the challenges involved in extracting information from historical texts which have been digitised.

In terms of the data heterogeneity problem, the Semantic Web (SemanticWeb, 2021), a vision of the World Wide Web Consortium (W3C), helps to build a web of data linked from different sources. Semantic technologies such as RDF, OWL, IRI and SPARQL have been proposed to enable data integration. Another valuable contribution from the Semantic Web is the use of ontologies which help to resolve the data variety problem by enforcing data standardisation and hence enabling the integration of heterogeneous data. A good introduction of Semantic Web is also provided by (Domingue et al., 2011). Ontologies are vocabularies that define data into a series of concepts and relationships that exist among those concepts. This definition creates a metadata layer that enables the information sitting in the data stores to be abstracted and represented in a standardized format. This then enables different data sources to be integrated through a metadata layer. Ontologies have been widely applied in bioinformatics and healthcare information systems. (Ison et al., 2013) shows the need to integrate information about bioinformatics operations through the use of ontologies. (He et al., 2017) presents innovative semantic-based methods to address important problems in healthcare. (Zhang et al., 2018) introduces an ontological framework to support integration of cancer-related data. (Sima et al., 2019) presents data integration and data enrichment using biological databases. On the other hand, authors in (Shbita et al., 2020) have published some geospatial data as semantic-rich linked data. They also used SPARQL queries to portray the changes in map segments, which provided better insight in spatio-temporal analysis tasks. Moreover, (Li et al., 2020) presents an approach to extract text content from historical map images and to generate a set of metadata that can be linked to a geospatial knowledge base. This linked data model then enables to perform complex queries for searching and indexing the historical maps.

In the field of ecology and conservation science, it seems imperative to collate heterogeneous datasets for better analysis. This highlights the scope for using ontologies to enable data integration. However, the complexity of integrating data is further compounded if data are provided from unstructured sources, such as the historical archives. Very few papers mention the combined use of Machine Learning, Natural Language Processing and Semantic Web techniques to build a comprehensive and meaningful data model based on information extracted from an unstructured source. These technologies not only hold the key to resolving the spelling variations faced with rule-based approaches but also to bringing to life and semantically-enriching information sitting in documents, which would have otherwise remain silo-ed for ever.

## 3 Data

This section presents a case study on the extraction of floristic information from a historical journal named the Journal of Botany (Britten, 1885), which we have used as a basis for experimenting with the proposed approach. This historical journal contains information on plant species observed in the area of Lake District over past three centuries. The information is considered very relevant to historians and conservation scientists as it gives a better sense of the floristic past and evolution in the Lake District.

Situated in the North West of England, the Lake District is a very popular tourist destination and is well known for its glacial ribbon lakes, rugged fell mountains and historic literary associations. In 2017, this region was inscribed as a UNESCO World Heritage (WHS) under the category of a ‘cultural site’. This category was established by UNESCO in 1992 to enable the identification, protection and conservation of sites deemed to be of ‘outstanding universal value’ and which were formed by the ‘combined works of nature and of man’ (UNESCOa, 2017/p. 19, UNESCOb, 1972/p. 2, Denyer, 2016/pp. 19–46). This UNESCO award has served to place renewed focus upon the best ways to conserve the region, which includes its unique flora; and has also served to link the region more closely to its recent historical past, in particular, on how the landscape has changed since the eighteenth century. This has led to the need for a better understanding of the historical past, and the natural and human processes that have shaped it over the past three centuries (Donaldson et al., 2017).

Due to its historical significance, the region has been described and celebrated in print by a wide variety of observers including naturalists, plant collectors, tourists, travellers, writers/poets (Donaldson et al., 2017). The UNESCO award has brought into focus the pressing need to better understand how the biodiversity of flora has changed from the historical past in order to better preserve and conserve. Critically, the datasets currently available to conservation scientists and policy-informing stake holders only extend back to the early 1960s as they principally rely on modern surveying techniques and quadrat survey data. Though there are some records before the 1960s, they are scant. A polygon query of the Lake District for ‘Plantae’ on the NBN Atlas (Atlas, 2021) resulted in 144490 matches, but only 11883 are dated before the 1960s and only 1643 before the 1900s. The lack of data pre-1960 makes it difficult for effective conservation strategies to be put into place. Historical sources can enable the investigation of the region's floristic past further back in time extending to the seventeenth century. This floristic information includes geospatial information about the different plant species found across the Lake District as well as information on their abundance and even on their habitats. However, the identification, extraction and correlation of such data from historical texts poses several technical challenges. Firstly, observations are scattered across a large body of text. To reveal the distribution of a single species across the whole region or the biodiversity at a specific site requires analysing and extracting data from a large volume of textual sources. Moreover, the language used to name and describe plants, and the places they grow, have both remained fluid over the past three centuries.

**Figure 1:**
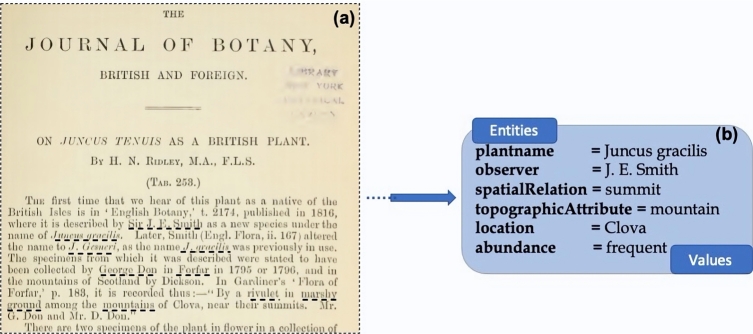
Types of information extracted from the Journal of Botany (figure (a) - courtesy of the Botany journal (1885)).

Journals like Flora of the English Lake District (Baker, 1885) and the Journal of Botany (Britten, 1885) are rich sources of information on flora in the Lake District. For this case study, we have looked at the Journal of Botany. Published in 1885, this journal contains a lot of information about plant species over time, places in the Lake District where they were observed, and people who have observed them and who have recorded their abundance in a particular region. An extract of the Journal of Botany is shown in Fig. 1 - (a) the underlined words show the kinds of information that need to be extracted; (b) highlights the types of information entities to be extracted from the Journal of Botany and an example of their values. The main information is around the plants that have been observed at a given location in the Lake District. There are also other relevant pieces of information such as the topographic attributes of a given locality, and the spatial relations of the plant species around those topographic attributes. For example, a particular plant named ***Juncus tenuis***, observed by ***L. Smith*** (observer), is ***common*** (abundance) at the ***bank*** (spatial relation of the plant with the topographic attribute) of a ***lake*** (topographic attribute) in ***Ambleside*** (location). Extracting such specific details as to where the plant was spotted can be very challenging, but can provide insightful information about the place and the plant.

## 4 Methods

This work is part of a cross-disciplinary project investigating the use of digital techniques to mitigate challenges faced in environmental science. We adopt an agile approach as the core methodology to underpin this research. An agile approach is one which is done in an iterative way, where each step can be revisited and altered as per the needs of a process. It encapsulates a range of principles and values expressed in software development. This approach helps to alleviate the inefficient traditional practices in software development where continuous meetings, heavy documentation, strict adherence to a Gantt chart used to be the norm. The result is a set of agile methods iterating through a continuous delivery of software in order to reach a solution. A good introduction to this approach is provided at (Ferrario et al., 2014). This agile process started off with a workshop where the inter-disciplinary project partners, researchers and stakeholders from the biodiversity community met together to reflect about the challenges involved in this domain. The proposed approach has been achieved in an agile way and has been applied to a case study presented in Sec. 3, which has been carried out by some of the researchers who came to the workshop. In this case study, they used rule-based approaches to extract information from a historical journal. However, they realised that this approach had some limitations and hence were seeking to know if there were any innovative digital technologies that can help to automate this process.

### 4.1 Proposed architecture

**Figure 2:**
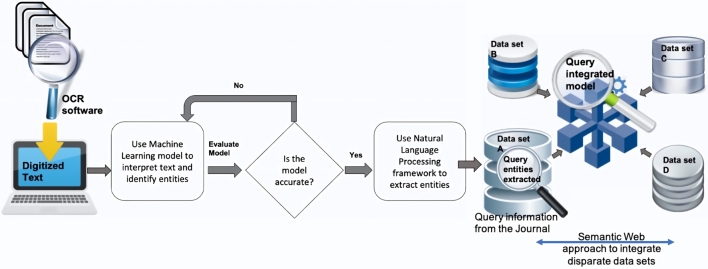
Methodology proposed to extract information from historical text.

The proposed approach is to automate the extraction of information from textual sources, enable the querying of this information and enriching these queries by integrating other data sources together with the information extracted. Fig. 2 depicts the pipeline presented in this proposed approach. The **first** step is to digitise a textual document if it is not in a digital form. This digitisation is achieved through an OCR-based software (used for Optical Character Recognition), which enables to identify text from scanned documents. The **second** step is to identify the different types of information contained in the document. This step involves the annotation of particular phrases from the corpus that represent the different information concepts. Once this annotation process is completed, we train a Machine Learning model to interpret these different concepts. An example of an ML model, in this case, will be to classify all the terms in the text that represent plant species. This is made possible through Natural Language Processing (NLP) frameworks which provide models to interpret the grammar of a given language and to recognise sequences of words from the text, which are referred to as **entities**. Common entities such as *‘people’*, *‘location’*, *‘country’*, *‘nationality’*, etc. can be recognised using some standard models available from most NLP frameworks. However, for custom entities, we need to train our own model. Depending on the requirements of the application, we can therefore use either a standard or a customised model. Once the entities have been identified, the **third** step is to extract this information using features such as Named Entity Recognition (NER) available from an NLP framework. The next section gives a brief overview of few NLP frameworks and their features. The **fourth** step is to then ingest the extracted information into a data store, shown as *‘data set A’* in Fig. 2. We propose a NoSQL database for this step owing to the schema-less nature, flexibility and scalability of NoSQL databases in interpreting unstructured information. This approach can enable scientists to query, to visualise the information extracted, and to also use the information in different applications. We can further enhance this approach, in a **fifth** step, by bringing the extracted information together with other related information from disparate sources in order to make richer queries. This is demonstrated in the form of *‘data sets B, C and D’* brought together with *‘data set A’* in Fig. 2, enabling to make enhanced queries from the unified model. The aim is to see if we can make more informed queries with different datasets combined together. For this step, we propose the use of Semantic Web techniques such as ontologies to integrate the heterogeneous data together and semantic data stores to load and query the integrated model.

### 4.2 Overview of technologies used

This section gives an overview of the technologies proposed in the methodology.

***Natural Language Processing frameworks*** Many Natural Language Processing (NLP) frameworks provide pre-trained models to help identify entities from textual documents, such as Stanford NER models^2^ and Spacy.^3^ Stanford NER provides good entity recognizers (also called models) for the English language, such as ‘person’, ‘organization’, ‘location’, and also recognizers/models for different languages. Stanford NER comes with well-engineered feature extractors for performing entity recognition and provides many options for defining feature extractors. Spacy is designed to facilitate the use of Natural Language Processing for interpreting and extracting text. The website of Spacy presents a list of its numerous features.^4^ Spacy provides some general-purpose pre-trained models to predict named entities, to recognise part-of-speech tags and to identify the syntactic dependencies between different components of a sentence. There are models available for a wide array of languages, which can either be used out-of-the-box or fine-tuned to recognize specific entities. Some of the custom entities identified by these models are *‘person’*, *‘nationalities’*, *‘buildings’*, *‘organisation’*, *‘countries’*, *‘time’*, *‘event’*, etc. These models can be further trained by the user in order to identify specific pieces of information. The purpose for adding labels to a word/phrase is to identify the word/phrase as a named entity, a process known as annotation. To create customised models, we need to annotate sequences of words with a label within a training dataset. This can be a tedious task especially if we have to do this programmatically. However, most NLP frameworks provide guidance on how to proceed in such a case.

***Prodigy*** Spacy provides an online annotation tool known as Prodigy^5^ for annotating text and for creating a custom model. Prodigy provides a continuous active learning system where the machine learns the annotations as we progress along. Prodigy enables to train and evaluate data so as to build machine learning models that can be used to recognize entities from text. This clever tool helps users explore their data, carry out error checks and develop some rule-based systems that can be used in combination with the models.

***MongoDB NoSQL database*** A NoSQL database is a type of database that stores data without the need to specify a relational schema of the data as is the case with relational databases. The flexible structure of a NoSQL database enables to easily store and query the extracted floristic data. For this case study, the data has been stored on MongoDB (MongoDB, 2021), a popular and easy-to-use NoSQL document-oriented database. To facilitate a visual query of the data stored on MongoDB, there are graphical tools available that can interface with MongoDB. One such tool is NoSQLBooster (NoSQLBooster, 2021) which provides an easy-to-use interface where queries can be formulated visually.

***The Semantic Web*** The Semantic Web (SemanticWeb, 2021) provides a common framework where data can be shared and reused across applications. One of the Semantic Web technologies is an ontology^6^ which models some aspect of the world (called a domain). Examples of domains can be an educational domain, a medical domain etc. The ontology acts as a vocabulary to explicitly define concepts, properties, relations, functions, constraints of a particular domain and also represents the schema of the data being modelled. It can also enable to uniquely identify each data concept through an IRI (Internationalized Resource Identifier). The use of the ontology is to semantically decorate or enrich data with concepts/relationships. The data can be labelled and defined using the concepts from the ontology, thereby providing an identification to the data. This unique identification mechanism, IRI, enables data to be standardized. This helps to resolve the heterogeneity problem that arises in data found in different formats. This enables to provide a layer of abstraction over the ‘raw’ data. For example, if we have two data sources containing information about plants observed at some locations, and if we enrich both using ontologies, then the datasets can be integrated through this standardization process. The World Wide Web Consortium (W3C) offers semantic web standards to create different forms of vocabularies/ontologies in a standard format, and enable the reuse of this data. Some of these formats are Resource Description Framework (RDF),^7^ RDF Schemas,^8^ Web Ontology Language (OWL).^9^ Moreover, there are standard ontologies, contributed by governing bodies, and also existing ontologies, contributed by the wider Semantic Web community, available for reuse. However, users can also create their own ontology if they have the required information to define a domain.

We have used Protege (Protege, 2020) software to create our ontologies. Protege is a well-established software and provides a graphical interface for the development of the ontologies. We used GraphDB (GraphDB, 2021) as our semantic data store to load the semantically-enriched data and to integrate the disparate datasets available and to query the information stored. GraphDB is a highly-efficient graph database providing support for semantic data (RDF) and enabling queries through SPARQL (Sparql, 2008), an SQL-like query language used for querying semantic data types. GraphDB can also support millions of triples. The data we have used was for a prototype; however, GraphDB is easily scalable and can be available as a cloud version to host data.

### 4.3 Automating the information extraction

This section gives more details on how the technologies (mentioned in Sec. 4.2) have been applied to automate the extraction and integration of information from the Journal of Botany.

The digitisation step had already been done through OCR-based software by the environmental scientists who provided the Journal of Botany in digital form. We document on the subsequent steps applied in the proposed methodology. In the initial stages of this case study, we tried experimenting with RapidMiner,^10^ a data science tool providing an easy-to-use graphical user interface for enabling the identification of entities from text. However, the models used by the tool (at the time of implementation) were trained to recognize a specific set of common entities. Hence, we resorted to using an NLP framework to customise our own model for entity recognition. Given the ease of use of the Prodigy tool and the fast performance of the Spacy NLP framework, we have opted to using Prodigy and Spacy to train a customized model to identify and extract the following entities from the Journal of Botany: ***plantname*, *observer*, *location*, *spatialRelation*, *topographicAttribute*, *abundance***. These tools evolve very quickly and their makers are often releasing their new versions. This is particularly the case with the Prodigy tool. At the time of implementation, the Prodigy version used was v1.6.1 and the current version at the time of writing was v1.10.5; Spacy was at version 2.0 and is now at v3.0. The newer edition of Prodigy provides more functionalities to error-check a model and to train a more accurate model. As for Spacy (v3.0), it provides similar functionalities as its previous counterpart but also provides more stability, performance and more experimental support for some new NLP innovative tasks. Nonetheless, the previous versions provided all the features necessary for model building and entity recognition.

The linguistic features provided by Spacy are shown in Table 1. The prominent features of Prodigy are *Named Entity Recognition (NER), Text Classification, Dependencies & Relations, Computer Vision, Audio & Video and Evaluation*. The main feature used in the prototype design is the *Named Entity Recognition* which enables to extract labelled pieces of information (entities) from text. Moreover, Prodigy also provides a bundle of built-in features to annotate text, train a model or compose models together to build more complex systems, and to error-check the model. The beauty of the Prodigy tool is that it enables the model to learn gradually based on the feedback provided by the user during the annotation process. Unlike other tools, Prodigy is a tool that can make suggestions to users regarding the annotations chosen by the user. This case study uses the *Named Entity Recognition (NER)* feature from Prodigy to identify named entities from the Journal of Botany. This feature can be summed up in 3 simple steps: (i) annotate raw text; (ii) train a model on the training data; (iii) enable the user to manually correct suggestions made by the model. The main NER functionalities available in Prodigy (v1.6.1) for this work are shown in Table 2. These features have been updated in the current version and their documentation is available on the Prodigy website.^11^ The design pipeline, presented in the subsequent subsections, is based on the architecture diagram presented in Fig. 2.

**Table 1:** Linguistic features of Spacy.

Features	Description
Tokenization	splits a text into meaningful segments, called tokens
Part-of-Speech	parses and tags a token as a component of the grammar (e.g. proper noun, adjective, etc.) and enables to make a prediction of which tag/label is more applicable in this context
Named Entity Recognition	assigns labels to contiguous spans of tokens
Entity Linking	assigns a unique identifier to each entity to perform entity linking
Merging & Splitting	enables merging and splitting of tokens
Dependency Parse	parses a sentence into different components like nouns, verbs, etc., and enables navigating through these components in a tree-like structure
Sentence Segmentation	splits sentences

**Table 2:** NER features used in Prodigy (v1.6.1).

Features	Description	Function name
Fully manual	Manually Annotate raw text	ner.manual
Add suggestions from patterns and update existing model	Use pattern files to annotate part of the text and manually add further annotations and add new entity to the existing model	ner.teach
Train the model	Train the model using the training data	ner.batch-train
Error-check the model	Check and manually correct the suggestions made by the model	ner.make-gold

### 4.4 Creating training and test datasets

The **first** step is to create a training and a test data sets from the digitized journal. Prodigy has been used to train the model and as part of its requirement, we need to create a training and a test set. The purpose is to train a Machine Learning model that can interpret the text from the Journal of Botany and identify the required entities. The training set is used to train the entity recognizer model whilst the test set is used to evaluate the accuracy of the model. The ratio of the training to test set is around 4:1. The training set consists of around 189 Word document pages (around 60,000 words) and the test set consists of 3 scattered short sections from the journal.

### 4.5 Annotating the text

**Figure 3:**
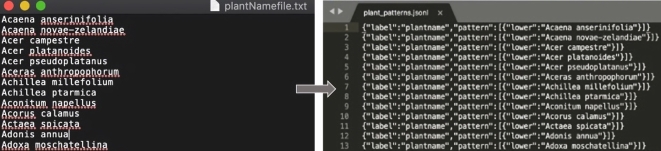
Creation of plant species pattern file.

The **second** step is to annotate the training data set with labels identifying the named entities *plantname*, *observer*, *location*, *spatialRelation*, *topographicAttribute* and *abundance*. Referring to the *NER* features provided by Prodigy (Table 2), we have used the feature *- Add suggestions from patterns and update existing model -* to annotate the raw text. One requirement for using this feature is to create a ‘pattern’ file which basically lists a sample of words or sequences of words that identifies a named entity. Prodigy uses this pattern file as a basis to annotate sequences of words in the training data set. Fig. 3 depicts the creation of a pattern file from a list of plant names for identifying plant names. As shown in the figure, the scientific names of plants consist of two parts - a genus name and a specific name. The pattern file (the figure in the right) highlights the format that the Machine Learning model should follow during the annotation process. For example, plant names consist generally of two words where the first character of both words is in uppercase and lowercase respectively and the entity for identifying plant names should be labelled *plantname*. This crucial step needs to be carried out for all the different entities that need to be identified from the text. For this, the environmental scientists compiled a list of different plant names, observer names, locations in the Lake District, possible terms to define a spatial relation, possible topographic attributes and common terms to define the abundance of plants. The pattern files were then created using a Python script.

### 4.6 Creating a Machine Learning model using Prodigy

The **third** step is to apply the ***ner.teach*** (see Table 2) function together with the pattern files to add new entity types to an existing model provided through Spacy (en_core_web_sm), a model for the English language. This model parses the English grammar and provides entity names for recognising some standard information. The ***ner.teach*** function is used to gather the most appropriate training data for a named entity recognition model by annotating phrases that syntactically match terms from the pattern files. The pattern files suggest the format of a given entity, and enable to annotate phrases that match this format (as shown in Fig. 3). Given that these pattern matches contain examples of phrases that can be annotated, they help the Machine Learning model by providing positive examples as a head-start. This third step also requires the interaction of the user with Prodigy, where the tool presents suggested phrases for a named entity and the user decides whether to **accept**, **reject** or **ignore** that phrase. Following the decision of the user, Prodigy will annotate the phrase using the ***ner.teach*** function. The existing model is then updated with new entities and is trained using the ***ner.batch-train*** feature (Table 2) on the annotated data set. Fig. 6 (a) shows an extract from the journal and Fig. 6 (b) shows the text after it has been annotated, highlighting the different entities. On the other hand, Fig. 7 portrays the Prodigy tool in action whilst annotating the text. Fig. 7 (a) shows how annotation of the text is carried out where by the function ***ner.teach*** is being used. The interface provides accept, reject and ignore options (green, red, grey boxes at the bottom of the screen) which we can use to accept, reject or ignore the annotations provided by the model. As we progress, the model learns along and provides further suggestions for the entity name. Once the model has been sufficiently trained, the model can be generated through another function called ***ner.batch-train***, and the model can be applied to identify named entities from a given text.

On the other hand, it is highly likely that the model may not have fully learnt to properly recognise the entity with the suggestions provided through ***ner.teach***. Fig. 7 (b) highlights an interesting feature of Prodigy where the suggestions made by the model (created in part (a)) can be corrected using a function called ***ner.make-gold*** (see Table 2). The word *‘Durrow’* is meant to be a location, which the model was not able to detect earlier. The ***ner.make-gold*** feature enables the user to manually annotate this word as a *Location* entity. Moreover, phrases which are annotated under a wrong label can also be corrected similarly. The model can thus be improved with a higher level of accuracy. The annotations that have been created are stored in a database by Prodigy, and they can be downloaded as a file and re-used for building other models.

### 4.7 Extracting the entities using Spacy NLP framework

Once we have our model, we can use an NLP framework to extract the entities identified from each sentence of our test data. For this purpose, we used the Spacy framework (see Sec. 4.2) to extract the entities so that they can be stored on a database and queried. We also used Spacy to understand the dependency of the entities with respect to other words in a sentence. The Spacy features used are ***Named Entity Recognition*** (NER) and ***Dependency Parsing*** (DEP) (see Table 1).

***Using NER and DEP*** The ***Named Entity Recognition (NER)*** feature is used to extract the entities identified through Prodigy whilst the ***Dependency Parsing (DEP)*** feature builds a dependency tree for navigating through the various components of a sentence. The dependency tree highlights components of a sentence like a head, subject nodes, object nodes, children of a particular node, verbs, nouns, etc. We found that only extracting entities using the ***NER*** is not enough; we need to extract some contextual information regarding those entities, and this is possible through the ***DEP*** functionality. This feature is particularly useful in understanding the relationship between the different parts of a sentence.

**Figure 4:**
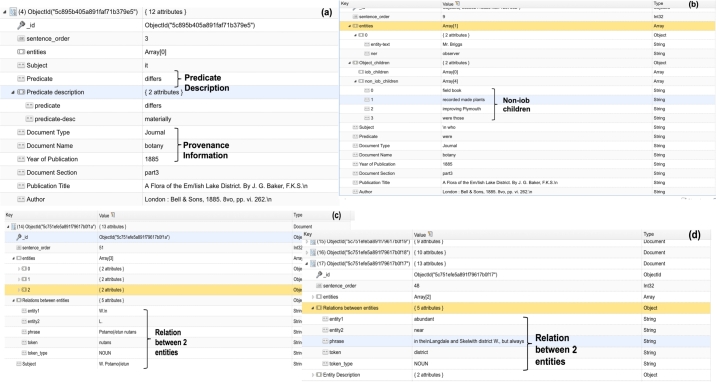
MongoDB queries showing entities extracted together their contextual information.

We wrote a Python script applying the *NER* and *DEP* functionalities to extract the entities and the relationship of each entity with neighbouring words from a given sentence. For example, *‘near’* is classified as a *‘spatialRelation’* entity in the text. Any noun appearing before an entity was classified as a descriptive noun to the entity. For example, in the phrase *‘remarkably near’*, *‘remarkably’* provides a description to the entity *‘near’*. Furthermore, if there are multiple entities in a sentence, we can find the relation between any two consecutive entities, and this is usually in form of a verb or a noun. We can extract the subject, relation/predicate and object of a sentence. If there is any adverb following the predicate, it is considered as the predicate description. The object, on the other hand, can have further children which can either be entities or non-entities. Fig. 4 gives an overview of these different contextual pieces of information surrounding an entity. Fig. 4 (a) shows the description around a predicate; whilst the predicate reads ‘differs’, the description gives more insight on this predicate (‘differs materially’). Fig. 4 (a) also shows the type of provenance information that has been added to the information extracted, such as *Document Type, Document Name, Year of Publication, Document Section and Publication Title*.

***Multi-word Entities*** Spacy also offers an IOB feature *(Inside-Outside-Between)*, which indicates whether an entity starts, continues or finishes on the tag used to represent that entity. This feature is particularly relevant for entities consisting of multiple words, for example, a phrase like ‘Orchis rnoriu’, annotated as a plantname, consists of two words. Using the Spacy IOB feature, ‘Orchis’ and ‘rnoriu’ are labelled as ‘B’ (Beginning of entity) and ‘I’ (Inside of entity) respectively. Any word before/after this entity is labelled as ‘O’ if it is not present in any other entity tag. The IOB feature helps to locate which words appear outside the entity, and which words form part of the entity, and also enables to provide accurate results especially when searching for the relationships of entities with other words in a sentence. Applying the IOB feature ensures to retrieve the appropriate entity for performing the dependency parsing. On the other hand, Fig. 4 (b) shows that an object can have children which can either be entities themselves or non-entities. In this case, the non-iob children refer to the object children which are non-entities. Fig. 4 (c) and (d) show two separate queries regarding the relation between two consecutive entities.

### 4.8 Querying the extracted information

The extracted information can be loaded onto MongoDB NoSQL database. The query results depicted in Fig. 4 come from MongoDB and the query interface is the NoSQLBooster interface. These queries have been described as the contextual information surrounding entities in the previous section.

### 4.9 Creating a linked data model using Semantic Web techniques

**Semantic enrichment of data** The extracted data can be visualised and queried, as shown in the previous section, and can be used in different applications. For instance, we worked with environmental scientists who wanted to use this data for a GIS application, where plant names, extracted from the journal, can be plotted on a map highlighting the locations in the Lake District where they had been seen. In order to do this, they needed to combine geocoordinate data with the names of the locations extracted, then plot the required data. They also had geocoordinate data for all the locations in that area, but these were stored in a separate dataset, and they needed a way to combine this data together with the extracted data on the plant names and their locations in order to create a map of the species at different locations. On the other hand, the scientists also had some other floristic information such as the taxonomy of plants and multiple synonym names of the plant species that they thought would be good to incorporate together with the extracted floristic information. All this information was stored in separate data sets. In order to facilitate this process, we proposed the idea of building a linked data model, which enables the integration of heterogeneous datasets into a unified model. Information can then be queried from this multisource data model and used in different applications. It should be noted that the extracted data can also be used in its primitive form. The purpose of adding the semantic layer is to highlight a new dimension that can be brought to enrich data with metadata and to query data, provided from multiple sources, from a unified model. The linked data model provides the possibility to run enhanced queries on the floristic information extracted from the Journal of Botany.

**Figure 5:**
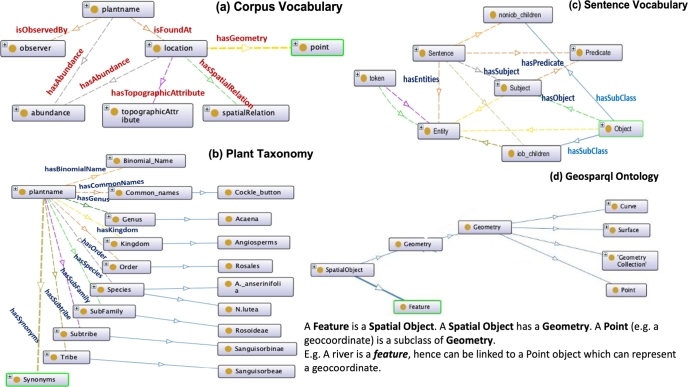
Vocabularies for the linked data model.

As explained in Sec. 4.2, ontologies are vocabularies used to define a domain through concepts and relationships. They are also used to resolve the heterogeneity arising in datasets by abstracting over the raw data and enabling an integration of disparate datasets. For this case study, we have created 3 ontologies: a corpus vocabulary, a plant taxonomy and a sentence vocabulary; and we have used the OGC GeoSparql ontology,^12^ which has been contributed by the Open Geospatial Consortium (OGC). The OGC GeoSPARQL ontology is a standard ontology that supports representing and querying geospatial data on the Semantic Web. The software used to create the ontologies is called Protege.^13^ The semantic enrichment has been carried out using a Python script. Fig. 5 highlights these 4 ontologies. The corpus vocabulary in Fig. 5 (a) shows a vocabulary defining the entities extracted from the historical journal (plant name, observer, etc.). The vocabulary also defines the relationships among the entities, for instance, a plant is observed by an observer; a plant is found at a location; a location has a coordinate point, etc. Fig. 5 (b) shows another vocabulary defining the plant taxonomy, highlighting the various terminologies that represent the family of plant species such as the *Genus, Family, Tribe, Synonyms, etc.* This vocabulary can be used to enrich plant names with metadata about their taxonomy. Fig. 5 (c) demonstrates another vocabulary that defines a sentence structure - a sentence has entities, a subject, a predicate and an object. The subject can be an entity whilst an object can have children which can be either entities *(iob_children)* or non-entities *(non_iob_children)*. This vocabulary is used to define the different components of a sentence that have been extracted to enhance the contextual information of the entities. Fig. 5 (d) shows an extract of the *GeoSparql* ontology in order to define a location having a geocoordinate value. An example of how this ontology is used is shown in this figure itself.

These vocabularies provide a layer of abstraction over the ‘raw’ data where they are used to enrich data with definitions. The data can be labelled and defined using the concepts from the vocabularies. For example, a plant like *‘Juncus tenuis’* is defined as a ***plantname*** concept and observed by *‘Smith’*, defined as an ***observer*** concept, near a *‘River Esk’*, defined as a *location* concept, in *Lake District* from the corpus vocabulary. This river, in turn, can be defined as a ***spatial object*** concept having a geocoordinate location from the *GeoSparql* ontology (see example in Fig. 5 (d)). The taxonomy of this plant can also be defined using the concepts from the plant taxonomy. Finally, the sentence vocabulary can be used to define the ***subject, relation and object*** concepts of a sentence where the plant *‘Juncus tenuis’* was retrieved. This semantic enrichment results in bringing together data from heterogeneous sets (plant synonyms data, geocoordinate data and the Journal of Botany) and at the same time, enriching the integrated data with metadata (such as plant taxonomy and contextual information regarding the sentence components). The advantage of this approach is that it is scalable and allows to bring together other datasets to this linked data model.

**Using a semantic data store** This semantically-enriched data can be loaded onto GraphDB for querying and visualising. SPARQL is used to formulate queries over the information stored on GraphDB. Since the information is provided from heterogeneous sources, GraphDB acts as a unified data model encompassing data from multiple sources. The queries executed have been presented in Fig. 9.

## 5 Results

This section presents an evaluation of the proposed approach in order to address the 3 research questions mentioned in the Introduction section. This section is broken down in 2 parts - assessing the NER model and assessing the linked data model.

**Assessing the Named Entity Recognition model** We consider the first research question here: to what extent can we extract information from unstructured historic texts? This is addressed by assessing the accuracy of the *Named Entity Recognizer (NER)* model, developed using the Prodigy tool, to see if all the required entities were identified from the Journal of Botany. These entities are *plantname, observer, spatialRelation, topographicAttribute, location and abundance*. The NER model has been trained to recognise all these named entities. These entities are then queried together with their contextual information - such as the predicate description, the relation between two consecutive entities and the provenance information - from the MongoDB data store where they are stored.

We used one chunk of the text from the journal for training the model and took 3 smaller chunks from different portions of the journal to test the NER model. Table 3 provides a rough estimate of the size of the training data and test data sets. Fig. 7(a) shows an extract of the training set and Fig. 7(b) shows the output of applying the NER model on one test data set.

**Table 3:** Training and Test data sets.

Data used in NER model	Size
Training Data	30000 words
Test Data 1	300 words
Test Data 2	400 words
Test Data 3	500 words

Each entity was initially identified individually and a separate model was trained for each entity. A composite entity is one that consists of 2 or more simple entities. To train a model to recognise such a composite entity, the annotations for each of the separate entity models were first merged into one dataset and then the model was trained on the merged dataset. The training of the composite entity model is more challenging as it requires the model to identify different pieces of information at the same time. This can easily lead to confusing outcomes especially where the model is required to identify ‘similar-looking’ phrases as being different entities. This is particularly the case with the plant names and the observer names. Both have got initials and consist of more than one word.

The complexity of each particular entity seemed to influence the accuracy of each respective model. The metrics used by Prodigy to calculate the accuracy of the model were based on the size of the text that was annotated, number of accepted, number of rejected and number of ignored annotations. The legend of the figures, depicting different colour schemes for accepted, rejected, ignored annotations and number of annotations, is shown in Fig. 8 (h). The accuracy was found to be low if there was a lot of rejected or ignored annotations over the number of accepted annotations. Therefore, we need to balance these metrics in a smart way; however, if the text itself is tricky (as is the case with the Journal of Botany), then it is a very challenging process. Table 4 shows these metrics for all the NER models that have been trained.

**Table 4:** Metrics for accuracy of the NER model.

Entity	Annotations	Accepted	Rejected	Ignored
plant	2645	489	54	2102
observer	3821	645	3176	0
abundance	1507	254	1253	0
spatialRelations	1247	312	935	0
topographicTerms	1247	312	935	0
location	3953	1179	2774	0
location_terms (spatialRelations+topographicTerms+location)	2501	390	67	2044
lakedistrict	13970	2820	5946	5904

The model was initially trained using the ***ner.teach*** feature and if the model was found to have a low accuracy, it was corrected using the gold training feature ***(ner.make-gold)***. In either case, the user is given the possibility to accept, reject or ignore a phrase/word annotated by the Machine Learning model. Prodigy enables users to store the annotated text in a database provided through Prodigy itself. The model is then compiled using the database of annotated text. The advantage is that we can re-use the set of annotations to train some other model or we can merge different such sets especially where we have to identify multiple entities from text. Therefore, we first trained a separate model for each individual entity, creating a database storing annotated text for that particular entity.

Except for ***‘abundance’***, ***‘spatialRelation’*** and ***‘topographicAttribute’*** all the other entities in this case study are quite complex. The text related to ***abundance***, ***spatialRelation*** and ***topographicAttribute*** are quite simplistic in nature such as ‘common, frequent, rare, scarce’, ‘near, borders, opposite, next to’ and ‘cliff, moor, fields, rocks, mountains’ in respective order. These entities were mostly represented using one word-text, which facilitated the training of their model compared to that of the more complex entities. As a result, we found that it was enough to train the ***abundance*** model using the function ***ner.teach*** only and to get an evaluation of 0.947 (Fig. 8 (c)). Similarly, ***spatialRelation*** and ***topographicAttribute*** were also trained using ***ner.teach*** only. Fig. 8 (d) and Fig. 8 (e) respectively show the accuracy of the ***spatialRelation*** model to be at 0.8 and that of the ***topographicAttribute*** model to be at 0.77. The ***location*** model (Fig. 8 (f)), used to identify different location names in the Lake District, was trained twice using ***ner.teach***. The models had an accuracy of 0.725 and 0.706 respectively. The annotated data sets from both iterations were merged into a separate dataset, which was then trained again similarly. The aim was to see if training a model which merges the output of 2 separate models could yield a more accurate model for a given entity. However, the accuracy of the resulting model was *0.622*.

Given that the ***spatialRelations*** and ***topographicAttribute*** entities normally accompany the ***location*** entity in sentences, we merged the annotated datasets of these entities. We then trained a model, based on the merged annotations, for a complex entity called ***locationTerms*** to identify *spatialRelations*, *topographicTerms* and *location* together. We wanted to see how efficiently the ***locationTerms*** entity model can identify the 3 entities together. The *locationTerms* was trained 3 times. The ***locationTerms_model_v1*** model reached an accuracy of *0.611*, whilst in the second iteration, the ***locationTerms_model_v2*** model was at *0.548* and finally the ***locationTerms_model_v4*** model was at *0.8*, as shown in Fig. 8 (g). The high accuracy value of *0.8* of the model in the last iteration is due to the fact that this model was a result of the corrections made to the model from the previous iteration using the gold training feature. This model was quite complex as it was trained to recognise three entities together, that is, ***spatialRelation, topographicAttribute and location***. One reason for a low accuracy value in the v1 and v2 models is owing to the complexity of the ***(spatialRelation-topographicAttribute-location)*** entities. The use of multiple words or even abbreviations to represent entities contributed to a lot of ambiguity in the models. Another problem encountered was that all three entities were not always present in a particular text, and also the model missed to recognise a ***spatialRelation*** or a ***topographicAttribute*** around a given ***location*** entity, which contributed to a low accuracy value.

Moreover, plant names have been trained mostly as consisting of 2 words (as per the binomial nomenclature) consisting of a genus name followed by a specific name, such as ‘*Acaena anserinifolia’*, *‘Lathyrus odoratus’*. However, there are cases where one of these names is written as an initial, for example, *‘L. angustifolia’*. Observer names can also have their first names written as initials either before or after the surname, or they can have their names written in full. Examples of observer names are as follows: *‘Atwood, M. M.’*, *‘J. G. Smith’* or *‘Broome, Christopher Edmund’*. This contributed to a lot of ambiguity surrounding plant names and observer names. Whilst training a separate model to identify the plant names, we decided to use the gold training feature to make any corrections to suggestions made by the model. Fig. 8 (b) shows a noticeable increase in the accuracy of the plant model from *0.624 to 0.827* through the gold training feature. On the other hand, Fig. 8 (a) shows that the accuracy of the observer model was at *0.60*. We did not re-train the model using the gold feature here.

The final model (called ***lakedistrict_model***) was a product of the merge of the annotated datasets for all the entities ***(plantname, observer, spatialRelation, topographicAttribute, location, abundance)*** and was trained around 8 times. In each iteration, we were correcting the model suggestions, using the ***ner.make-gold*** training feature. This became necessary especially when the model was required to identify so many entities, and the probability of error was anticipated to be high. This was found to be particularly relevant for the plant names and observer names. The use of the ignore feature, however, seemed to contribute to a better accuracy of the model especially when we annotated a significant portion of the text. Fig. 8 (h) shows the accuracy of the final three models to be *0.779, 0.731* and *0.714* respectively. The models were corrected in each iteration using ***ner.make-gold*** in order to get a better accuracy.

Regarding the querying of the entities, it is possible to query the entities along with their contextual information. As shown in Fig. 4, these contextual details range from the provenance information (see (a)) through to any relationship between 2 entities found in a sentence (see (c & d)) to any other contextual information termed as iob/non-iob children (see (b)). The extra pieces of information represent any dependencies between the entities and other phrases extracted from a sentence.

**Assessing the linked data model** This section aims to answer the other two research questions: to what extent can we integrate the extracted information with other information from a range of disparate sources?; to what extent can we then gain scientific insight from querying across the resultant integrated information model?

These research questions are addressed through an evaluation of the linked data model that has been used to integrate disparate data sets. The linked data model was assessed to see if we can query for information found in the individual datasets and also to see if we can query for information sitting in these datasets that have been linked together. This unified model was hosted onto the GraphDB store, and was queried using the SPARQL capability of GraphDB. To create this linked data model, we brought in the following information in order to enhance the queries made on the floristic information: (i) plant taxonomy: plant names extracted from the journal can be tagged with their taxonomy; (ii) plant synonyms: plant species names extracted from the journal can be accompanied with their different existing synonyms; (iii) plant metadata: information concerning a plant species name such as the description of the plant; (iv) geocoordinates of locations: the geocoordinate information, linked to the locations where the plants have been found. This integration is particularly useful if the extracted information will be used in applications, such as the GIS application mentioned earlier, where the plant names need to be plotted on maps based on the locations where they were found. Not to mention that we can bring in other plant-related data sets in this model if available.

Fig. 9 demonstrates a few queries executed from this unified model. Fig. 9(a) links the document to a synonyms dataset so that we can also retrieve the synonyms of the plant species names that have been extracted. Fig. 9(b) shows a dataset storing plant taxonomy data; hence, plant names extracted from the journal can be tagged with their taxonomy using this dataset. Similarly, Fig. 9(c) shows results of a query made against the historical texts and a dataset containing plant metadata such as a description of the plant, a binomial value, etc. Through such a query, the user can also get more information concerning a plant species name. Finally, Fig. 9(d) shows how a dataset about geocoordinates of locations is linked to the plant names found in particular locations, and how we can tag the locations extracted from the journal with their geocoordinate information.

The difference between MongoDB and GraphDB queries is that MongoDB queries are made against one data set whereas GraphDB queries are made against a unified model combining disparate data sets together. The MongoDB queries (see Fig. 4) reflect only the data extracted from the Journal of Botany, which revolves around the entities found and some contextual information around these entities. On the other hand, the GraphDB queries are able to demonstrate any additional information about the entities extracted by bringing in information from other information sources. As demonstrated above, with GraphDB, we can query for other information alongside the entities such as: synonyms of a plant name, taxonomy of plant species, geographical coordinates of locations - all of which not found in the Journal of Botany. This list is not exhaustive as we can bring in other datasets, if available, to this linked data model. For example, we can bring in more information on the observers, on the historical significance of the Lake District, etc. We can even bring in people's views on the different regions of the Lake District. The purpose of the linked data model is to enrich the queries made by bringing in more information. This can provide more insight to scientists who are studying the region and its species.

As a matter of fact, the linked data model can grow bigger; however, most semantic stores are quite scalable. GraphDB is designed to support millions of rows and can even provide a cloud storage to accommodate large datasets. For our prototype model, although the data used was on a small scale, there were lots of triples that were generated. Nonetheless, the time taken for running the queries on GraphDB was trivial and ranged from 0.1 to 0.3 seconds, as shown in Table 5. Querying the datasets individually takes around 0.1 s while querying across more than one dataset takes up to 0.3 s. The linked data model was also accommodated on GraphDB running on the host machine.

**Table 5:** Time taken to query from GraphDB (in seconds).

Query	Time Taken (s)
Query a sentence from the journal extract	0.1000 s
Query from the geocoordinates database	0.1000 s
Query from plant taxonomy dataset	0.1000 s
Unified Query from journal extract and synonyms dataset	0.3000 s
Unified Query from journal extract and geocoordinates dataset	0.2000 s
Unified Query from journal extract and taxonomy dataset	0.2000 s

## 6 Discussion

**Figure 6:**
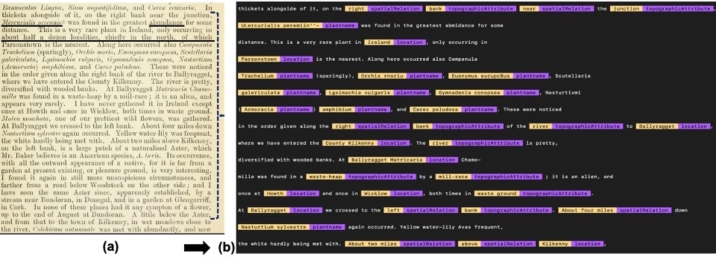
Annotated extracts from Journal of Botany (figure (a) - courtesy of the Botany journal (1885)).

This paper has shown an approach to automate the extraction of information from a historical text, to query such information together with their contextual details and also to bring this information together with related information from other data sources in order to enrich the queries. However, there are different factors that need to be taken into account when applying Machine Learning and Natural Language Processing techniques to train a Named Entity Recognition model to recognise entities from text. Firstly, the quality of the text plays an important role in determining the accuracy of the NER models, for example, the likelihood of having mis-spellings in words is higher in a paper document that has been digitized. The quality is even more compromised with dated text as is the case with the Journal of Botany. The fact that we looked at a historical document meant that some OCR errors were encountered. To properly interpret the spelling errors, created during the OCR process, is a challenge. Although the majority of text seemed to be properly digitized through OCR, nonetheless, there seemed to be few discrepancies. Referring to Fig. 6 (a), the underlined phrases highlight the text-related challenges that need to be resolved first before we train a Machine Learning model. The first underline shows a plant name which should read as *‘Mercurialis pernnis’*. However, it was interpreted as a completely different name in Prodigy (*‘ULercurialis peremin”-’*). The second underline shows a word ‘abundance’ which has not been read properly by Prodigy and instead appears as ‘amindance’ (see Fig. 6 (b)). The third underline is completely missing in the Prodigy output. Hence, we need to carry out some pre-processing on the data before actually training any NER model. On the other hand, the journal Flora of the English Lake District (Baker, 1885) seems to be more structured than the Journal of Botany. It would be interesting to run this approach using this journal instead and compare the results.

**Figure 7:**
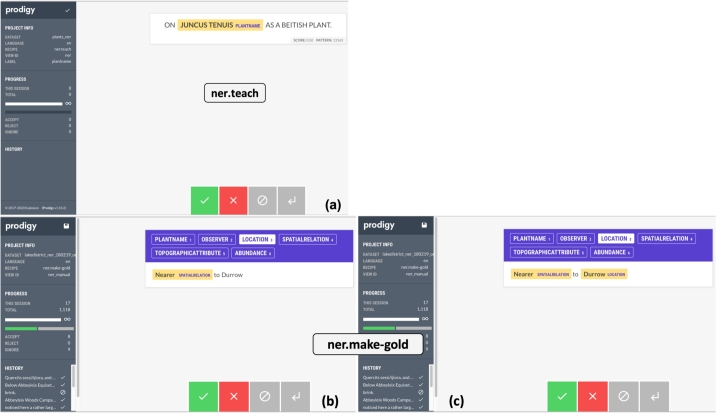
Annotating extracts from Journal of Botany using Prodigy.

**Figure 8:**
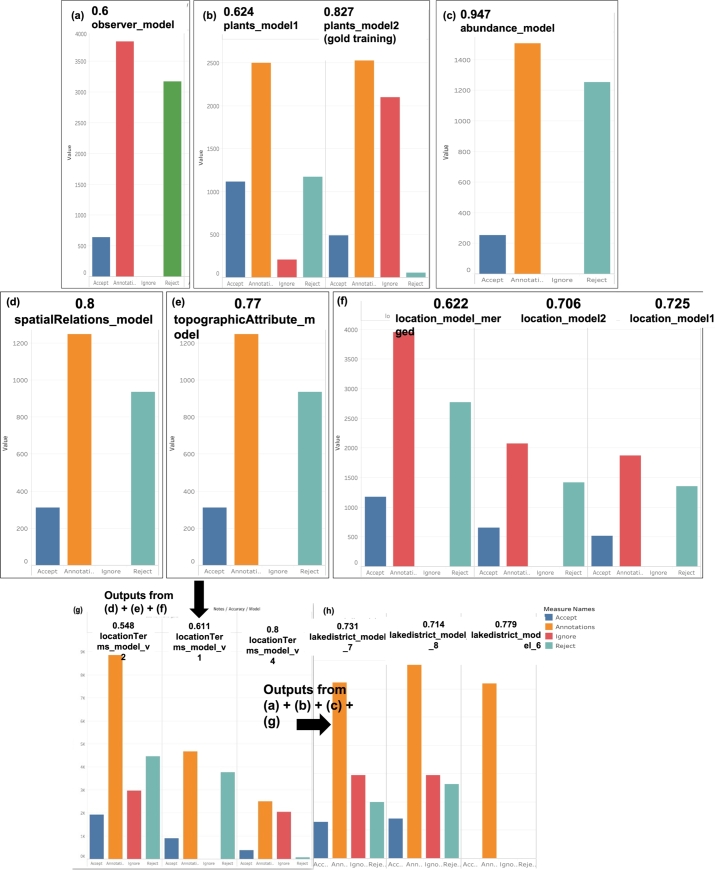
Evaluation of the NER model trained in Prodigy (v1.6.1).

**Figure 9:**
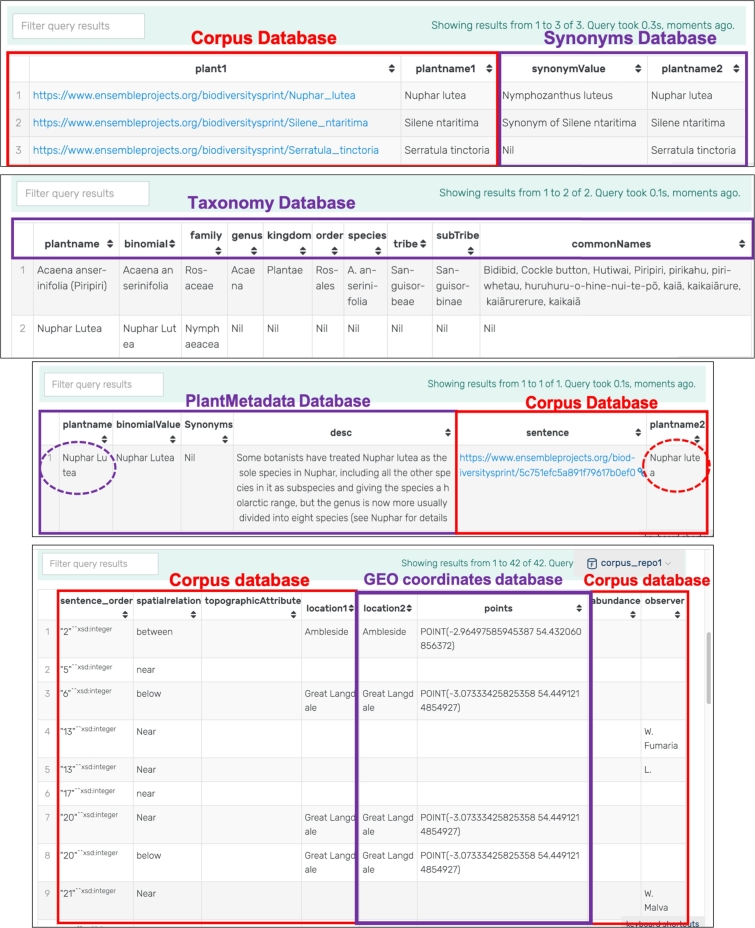
Querying the linked data model on GraphDB.

The content found within a document also plays a role in determining the accuracy of the model. The more complex an entity is, the more challenging it is to train the model to properly identify the entity. This causes some discrepancies in training a model to properly identify phrases that may look similar but represent totally different entities. This is the case for plant names and observer names, which seem to have a similar format in the Journal of Botany. For instance, plant names and observer names are represented using multiple words and even initials such as *J.Gesneri* or *H.C. Hart*, which made it difficult to tell if it is a plant name or an observer name. Moreover, OCR errors occurred during the digitization process meant that there were some additional characters added to some names. For example, *J.Smithii* was classified as a plantname when it is an observer name. On the other hand, certain outliers have been noticed concerning the plant names, for example, *Campanula Trachelium* is a plant name where the second word starts with a capital letter unlike other plant names where the second word is in lowercase letters. Hence, this particular outlier was not identified as a plant name. Therefore, we need to train the *plantname* model with different plant name representations, either in the form of the outlier or in the form of initials appearing in the names. We also need to train this entity with the synonyms of the plant names. Some synonyms are represented as *S.campetris*, a format that does not match the pattern used to train the plantname model (see Fig. 3). Given the syntactic similarity in the *observer* and *plantname* entity representations, it became very challenging to train a model to accurately identify these entities together. Some phrases which were meant to be a plant name, were recognised as observer names. This caused a lot of ambiguity during the training phase where we had to train the model to recognise these entities together. Initials compounded this complexity further, and as a result plant names having an initial to represent their genus name, were interpreted as an observer name. This is because the pattern files (see Fig. 3) defined the format of plant names to be explicit as two separate words, and the format of observer names to be the same but also to accommodate initials. Moreover, a composite model such as the *LocationTerms* model developed to recognise entities like *spatialRelation*, *topographicAttribute* and *location* together is also quite challenging to train. All three entities may not be present in a text, hence lowering the accuracy of the model. It would make more sense to keep the models separate to identify each of these entities. Another issue was the frequent occurrence of the abbreviation ‘L.’ in the literature, which is a standard abbreviation used in botany for ‘Linnaeus’,^14^ telling the reader who first named the plant. The occurrence of this initial caused some ambiguity in training the model as they were being categorized either as an observer name or a plant name.

Given that we are dealing with a very bulky document, we could not possibly run our model on the entire document. We had to segment the document into different sections so that our model could handle one segment of data at a time. This segmentation can either be done programmatically or through data processing tools such as RapidMiner which provides a segmentation feature to divide a document into separate smaller files.

The SpaCy NLP framework performed well in extracting the entities using the *Named Entity Recognition (NER)* feature and in analysing the contextual relations of the entities with neighbouring words through the *Dependency Parsing (DEP)* feature. For the NER and DEP work, we analysed one sentence at a time. Regarding Prodigy, it was found to be a very efficient tool for training a custom model. For every entity, we trained an individual model which resulted into a dataset of annotations for the given entity. We then merged all the annotated datasets and trained the resulting model to identify all the required entities. The latest Prodigy release boasts about additional and more robust features to error-check and to train a model. Nonetheless, the Prodigy version used in this paper still produced solid results as shown. The use of Prodigy emphasizes that there are digital tools available that can help to shape how we think about and use data.

Regarding the linked data model, we can bring in other floristic information into the model. The aim is to show that more enriched queries can be made from a linked data model, showing different dimensions of data that would have otherwise probably remained silo-ed. The queries drawn from this model show the power of a linked data model in bringing together data from heterogeneous sources. The time taken to query the linked data model was trivial owing to the fact that the datasets used were comparatively small (around 4000 triples/records). However, GraphDB comes in different versions to handle small to big projects with varying query loads. It is one of the most popular graph databases to create a linked data model. GraphDB also provides cloud-based versions if we want to scale the linked data model to accommodate bigger data sets.

## 7 Conclusion

This paper has examined the potential role of Machine Learning, Natural Language Processing and Semantic Web techniques to extract information from an unstructured textual source and to integrate this information with some other information from disparate sources. Existing data integration approaches are mostly focussed on bringing structured information sources together. The paper highlights an approach to extract meaningful information from an unstructured source and produce information-rich queries. The paper experiments the proposed approach through a case study on extracting relevant floristic information from the Journal of Botany. The application of NLP techniques to extract data from texts relevant to the field of ecology and conservation science is still in its infancy and there is currently no significant contribution around using NLP to extract information from complex historical data sources in this area. The paper shows that it is possible to automate the extraction of information from text, even from a challenging text such as the Journal of Botany, and also demonstrates how to bring this extracted information together with information from other data sources. The linked data model also plays a pivotal role in enabling a richer querying of information from multiple data sources in order to provide better insight to the field under study. The linked data model also demonstrates how to bring to surface information that would have otherwise remained silo-ed. The combination of Machine Learning, Natural Language Processing and Semantic Web techniques demonstrates that it is possible to build such a linked data model bringing information from unstructured and structured sources together.

The case study presented in this paper required training Machine Learning models to recognise the following entities: *plantname, observer, spatialRelation, topographicAttribute, location, abundance*. Referring to the three research questions presented in the Introduction section, we have managed to extract all the mentioned entities through the proposed approach. We also showed how we can enhance the query results by integrating other related data sets to the extracted information. For example, enhancing the plant species information with plant taxonomy data or enhancing the location information with geographical details all seem to contribute to producing information-rich queries. Moreover, such queries can also serve as input to applications that can be built on top of the linked data model. For instance, the geographical details regarding a location can serve as input into a GIS application.

Moreover, we also learnt quite a few lessons along this whole process. We found that training a model with all the entities from the start had an impact on its accuracy, and found that assembling a complex model incrementally using simple models helped to preserve the accuracy of the final model. The final model was also trained progressively on different batches of the training data rather than training it on a big set of data, and in each iteration, the model was corrected using the gold training feature of Prodigy. We found that this gold training feature was particularly useful for training complex models as it allowed the user to correct the model suggestions. We also found that the ‘ignore’ feature was useful to balance the ‘accept’ and ‘reject’ outcomes as we think that having too many ‘reject’ annotations compared to ‘accept’ considerably lowered the accuracy of the model. But this can be subjective to the size of the text that has been annotated.

The quality of the text also plays a big role in determining the accuracy of a model. The approach has also highlighted a number of challenges when extracting information from text, such as mis-spellings in words, initials or abbreviated names, etc. These challenges need to be resolved first before attempting to train an accurate Machine Learning model to recognise custom entities. Data pre-processing is a necessary step to resolve these discrepancies before applying any Machine Learning model to interpret text. A model can be trained more accurately if the discrepancies, highlighted in Sec. 6, can be handled. This will ensure that the Machine Learning model can accurately identify information entities in the subsequent stages of the pipeline. It should be noted that information has been extracted from one sentence at a time from a given portion of the text. The context of the entities has been restricted to the sentence where the entities were found. However, it will be interesting to see if we can reason about the context of the entities in a given portion of text such as a paragraph. In this regard, we are investigating into ways to achieve this and also to resolve inconsistencies linked to the quality of the text as a follow-up to this work. As a future work, we also intend to better understand the significance of the information extracted.

Finally, we think that this approach is a significant step that can be applied to other disciplines as well. It can help pave the way to retrieve insightful information from medical records, criminal records, government documents etc. and can prove beneficial to the respective parties in drawing insights from data.

## Declarations

### Author contribution statement

Vatsala Nundloll: Conceived and designed the experiments; Performed the experiments; Analyzed and interpreted the data; Wrote the paper.

Robert Smail: Analyzed and interpreted the data; Contributed reagents, materials, analysis tools or data; Wrote the paper.

Carly Stevens: Contributed reagents, materials, analysis tools or data; Wrote the paper.

Gordon Blair: Analyzed and interpreted the data; Wrote the paper.

### Funding statement

This work was supported by 10.13039/501100000266Engineering and Physical Sciences Research Council [EP/P002285/1].

### Data availability statement

Data included in article/supplementary material/referenced in article.

### Declaration of interests statement

The authors declare no conflict of interest.

### Additional information

No additional information is available for this paper.

## Footnotes

2 https://nlp.stanford.edu/software/CRF-NER.html.

3 https://spacy.io/.

4 https://spacy.io/usage/facts-figures.

5 https://prodi.gy/.

6 https://www.w3.org/standards/semanticweb/ontology.

7 https://www.w3.org/RDF/.

8 https://www.w3.org/TR/rdf-schema/.

9 https://www.w3.org/OWL/.

10 https://rapidminer.com/.

11 https://prodi.gy/docs/named-entity-recognition.

12 https://www.ogc.org/standards/geosparql.

13 https://protege.stanford.edu/.

14 https://en.wikipedia.org/wiki/Binomial_nomenclature.
